# The Global Clinical Trial Landscape for Children and Adolescents With Cancer

**DOI:** 10.1001/jamanetworkopen.2025.52510

**Published:** 2026-01-06

**Authors:** Margit K. Mikkelsen, Linh T. D. Pham, Catrian Sotelo, Mackenzie Kelley, Michael Edwards, Ryan R. Lion, Hanna Ravi, Guillermo Chantada, John T. Lucas, Giles W. Robinson, Victor Santana, Meenakshi Devidas, Carlos Rodriguez-Galindo, Daniel C. Moreira

**Affiliations:** 1 Department of Global Pediatric Medicine, St Jude Children’s Research Hospital, Memphis, Tennessee; 2 Department of Oncology, St Jude Children’s Research Hospital, Memphis, Tennessee; 3 Pediatric Hematology and Oncology, Hospital de Pediatría J.P. Garrahan, Buenos Aires, Argentina; 4 Eastern Virginia Medical School, Norfolk; 5 Center for Cancer and Blood Disorders, Children’s Hospital of Colorado, Aurora; 6 Department of Pediatrics, University of Colorado School of Medicine, Aurora; 7 Pediatric Hemato-Oncology, Fundacion Pérez Scremini-Hospital Pereira Rossell, Montevideo, Uruguay; 8 Oncology Department, Hospital Sant Joan de Déu, Barcelona, Spain; 9 Department of Radiation Oncology, St Jude Children’s Research Hospital, Memphis, Tennessee

## Abstract

**Importance:**

The improvement of childhood cancer outcomes has relied on clinical trials, primarily from high-income countries (HICs). Inconsistent access to clinical trials, especially in low-income countries (LICs) and middle-income countries, contributes to disparate care and limits the generalizability of conclusions from trials conducted in HICs.

**Objective:**

To describe the global availability of clinical trials for children and adolescents with cancer in different economic settings.

**Design, Setting, and Participants:**

In this cross-sectional study, the World Health Organization’s International Clinical Trials Registry Platform was used to search for clinical trials on May 8, 2024. Trials were included if they were interventional studies for pediatric patients (<18 years old) with an active cancer diagnosis.

**Main Outcomes and Measures:**

Clinical trials were analyzed based on geographic location, World Bank country income classification, and trial characteristics, including study phase, sponsor type, intervention type, and inclusion criteria. The main outcome was differences between HICs and low- and middle-income countries in the availability, design, and results of clinical trials for children and adolescents with cancer.

**Results:**

Of 138 595 oncology trials, 5645 met the inclusion criteria. Of these, 3149 (55.8%) were pediatrics-only trials. Most pediatrics-only trials (2558 of 3149 [81.2%]) were conducted by sponsors in HICs. Among identified trials, 12.5% (394 of 3149) were open for accrual in upper-middle-income countries (UMICs), 8.9% (281 of 3149) in lower-middle-income countries (LMICs), and 0.2% (7 of 3149) in LICs. Compared with HICs, LMICs or LICs registered fewer early-phase trials (28.8% [42 of 146] vs 81.4% [1709 of 2100]; *P* < .001), trials with cancer-directed treatments (17.6% [46 of 262] vs 76.8% [1964 of 2558]; *P* < .001), and multi-institutional trials (6.5% [17 of 262] vs 54.2% [1386 of 2558]; *P* < .001). LMICs or LICs registered more supportive care–based trials (81.7% [214 of 262] vs 19.9% [509 or 2558]; *P* < .001). Supportive care trials, later-phase trials, and trials from UMICs, LMICs, and LICs were more likely to publish clinical trial results within 10 years.

**Conclusions and Relevance:**

In this cross-sectional study of global pediatric oncology clinical trials, low- and middle-income countries were underrepresented. These data illustrate the need to improve the clinical research infrastructure in low- and middle-income countries and foster inclusive collaborations to promote equity in global pediatric cancer clinical trials.

## Key Points

**Question:**

Where are clinical trials for children and adolescents with cancer conducted globally?

**Findings:**

In this cross-sectional study, a review of the World Health Organization’s International Clinical Trials Registry Platform identified 5645 interventional pediatric oncology trials, with 81.2% of trials located in high-income countries. Trials in low- and middle-income countries were more often conducted by single institutions and concentrated on supportive care, while a similar rate of publications was seen across all country income groups.

**Meaning:**

This study suggests that systematic efforts to strengthen the research capacity in low- and middle-income countries may improve care and expand the generalizability of interventional studies for children and adolescents with cancer globally.

## Teaser

This cross-sectional study uses the World Health Organization’s International Clinical Trials Registry Platform to describe the global availability of clinical trials for children and adolescents with cancer in different economic settings.

## Introduction

Clinical trials are essential to the improvement of outcomes for children and adolescents with cancer and have contributed to the more than 50% increase in overall survival and decreased morbidity among this group in high-income countries (HICs) over the past 5 decades.^1,2^ However, the design and implementation of clinical trials is complex and involves factors such as funding, regulatory oversight, mechanisms for safety monitoring and reporting, dedicated research staff, and reliable access to diagnostic and therapeutic modalities.^3,4,5^ This complexity makes clinical trial availability challenging in low-resource settings, where these factors may be limited.^6^ As 80% of the estimated 400 000 new cases of pediatric cancers annually occur in low-income countries (LICs) and middle-income countries, these challenges are especially problematic and limit the generalizability of the knowledge generated by clinical studies.^7,8,9,10^ The World Health Organization (WHO) Global Initiative for Childhood Cancer has called for expanded access to clinical trials as a step toward achieving the goal of a 60% survival rate for all pediatric patients with cancer by 2030.^1^ Equity in clinical trials worldwide is essential to more accurately reflect the diverse genetics and social determinants of health of patients with cancer.^5^

Regional and national collaborative groups have been crucial in developing clinical trials for children and adolescents with cancer.^11^ However, many low- and middle-income countries continue to lack representation in these groups. Although many presume that there is a significant lack of pediatric oncology clinical research in low- and middle-income countries, few analyses have quantified or characterized this presumption. Previous studies to evaluate disparities in clinical research have done so by characterizing research consortia or by analyzing clinical trial availability based on published literature rather than clinical trial registries.^2,11,12,13,14^ Furthermore, these studies have not been specific to pediatric oncology. Here, we analyzed the global landscape of clinical trials for children and adolescents with cancer by using the WHO’s International Clinical Trials Registry Platform (ICTRP).

## Methods

### Database Search and Coding

In this cross-sectional study, the ICTRP was used to query and capture information about registered clinical trials globally. The ICTRP was established in August 2005 and aims to provide a platform for the prospective registration of all clinical trials internationally. The ICTRP collates data from 18 clinical trial registry databases, including Clinicaltrials.gov, Chinese Clinical Trials Registry, Pan African Clinical Trials Registry, and Brazilian Clinical Trials Registry. Although the number of countries represented can fluctuate over time with new registrations, the ICTRP includes trials from countries in all 6 WHO regions.^15^ As this study involved publicly available, nonidentifiable data, no institutional review board submission was required. Based on St Jude Children’s Research Hospital policies, this work does not qualify as human participant research and does not need to be reviewed by the institutional review board. This study followed the Strengthening the Reporting of Observational Studies in Epidemiology (STROBE) reporting guideline.

The terms *cancer*, *tumor*, *neoplasm*, and *malignancy* were used to search the ICTRP on May 8, 2024. No specific date range was defined for the search to capture all trials in the registry. Disease-specific searches were also performed for 16 of the most common childhood cancers, including but not limited to acute lymphoblastic leukemia, medulloblastoma, pilocytic astrocytoma, neuroblastoma, and retinoblastoma. Duplicate trials were removed and the search output was filtered to include only interventional studies and those with an inclusion age of younger than 18 years. The remaining studies were manually screened with the following inclusion criteria: (1) the studies included an intervention (ie, diagnostic, therapeutic, or supportive care), (2) patients younger than 18 years of age were eligible, and (3) study participants had an active cancer diagnosis included in the International Classification of Childhood Cancer, version 3.^16^ For validation, 32 clinical trials for children and adolescents with cancer were selected a priori and were confirmed to be captured by the search criteria. These trials were selected to represent the range of pediatric malignant neoplasms and countries from diverse regions and income statuses (eTable 1 in Supplement 1).

Data extracted directly from the ICTRP included source registry, registration date, phase, title, inclusion ages, and inclusion diagnoses. Additional information was manually coded, including oncologic diagnosis by primary clinical groups, disease status, intervention type, primary sponsor type, patient accrual sites, and country status based on World Bank income group classification.^17^ World regions were defined according to the International Agency for Research on Cancer.^18^ Studies were coded as pediatric if they included cancers seen mainly in children and adolescents (eg, neuroblastoma or medulloblastoma). Clinical trials were coded as pediatric and adult if they included cancers that were seen predominantly in adults but can occur in children and adolescents (eg, melanoma or thyroid carcinomas). Trials that included only pediatric patients are referred to as *pediatrics only*. The combination of pediatrics-only trials and pediatric and adult trials is referred to as *combined trials*. All abstracted studies were reviewed by ^2^ study authors (M.K.M., C.S., M.K., M.E., R.R.L., and H.R.) for accuracy.

The identified clinical studies were then queried in PubMed and Google Scholar for corresponding published results using the trial identification number and title. Only results published in peer-reviewed manuscripts were included. The publication date was considered the date of the first publication.

### Statistical Analysis

Descriptive statistics were used to analyze all items; χ^2^ tests were used to compare patient accrual site, patient accrual country, intervention timing, intervention type, and phase across groups, and Fisher exact tests were used to compare cancer type and primary sponsor type across groups. Analyses were performed on both pediatrics-only and combined trials. Due to small sample numbers, comparative analyses were performed with LICs and lower-middle-income countries (LMICs) combined into 1 group. For these analyses, early-phase trials were those that were categorized as phase 0, 1, 1/2, or 2, while later-phase trials were those categorized as phase 2/3, 3, 3/4, or 4. The Wilcoxon rank sum test was used to compare the median time to publication and the Gray test was used to compare cumulative incidence rates of publication, over time by trial type, trial phase, and between HICs, upper-middle-income countries (UMICs), and LMICs or LICs. All *P* values were from 2-sided tests and results were deemed statistically significant at *P* < .05. Analyses were performed using R, version 4.4.1 (R Project for Statistical Computing).

## Results

A search of the ICTRP database yielded 138 595 studies. After removing duplicates and restricting by study type, participant age, and inclusion criteria, we included 5645 clinical study registrations in our analysis (eFigure 1 in Supplement 1). Of these, 3149 (55.8%) were pediatrics-only trials. The search captured trials registered between 1987 and 2024.

**Figure 1.**
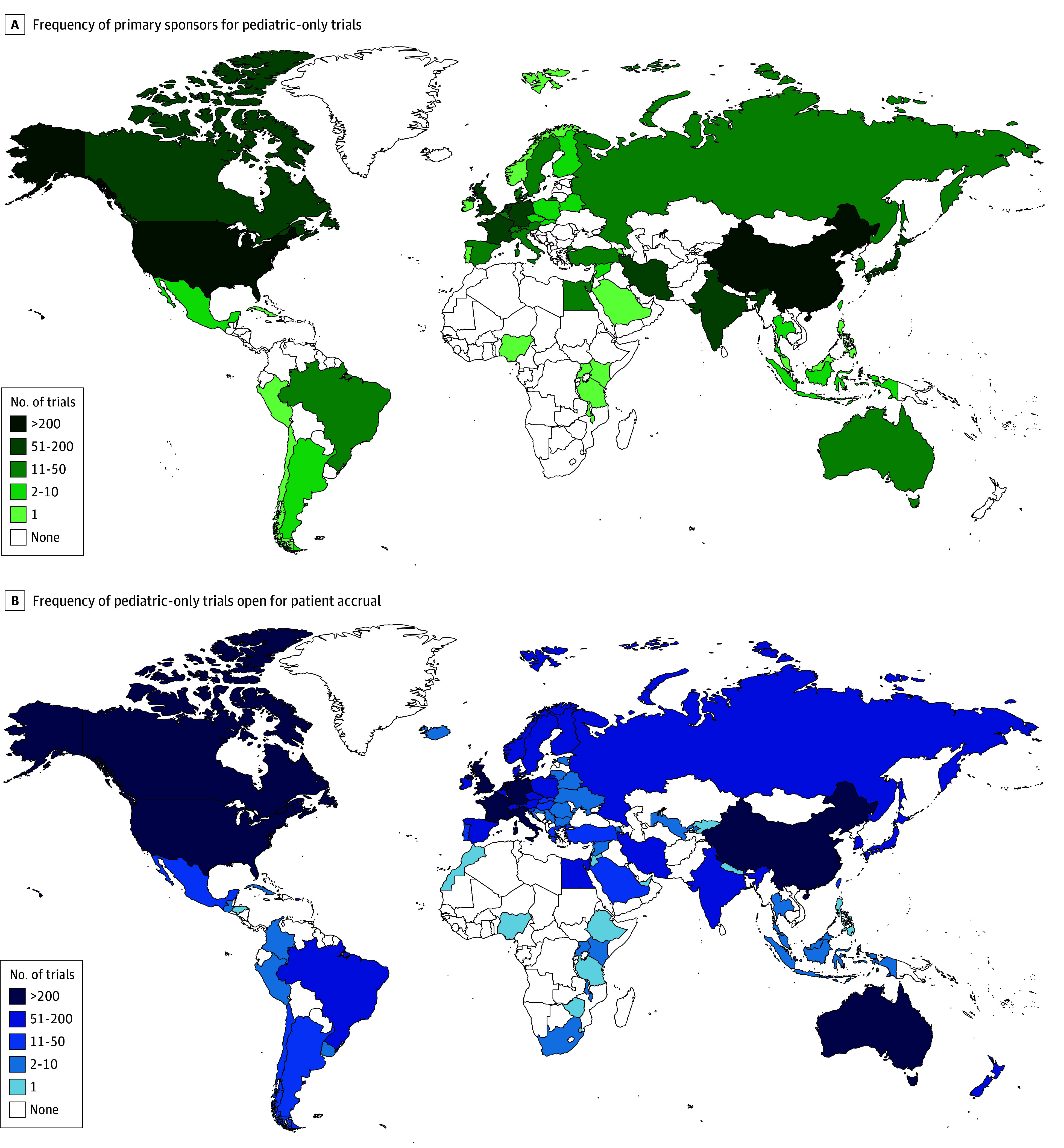
Geographic Distribution of Primary Sponsor and Patient Accrual for Clinical Trials A, Heat map of primary sponsor frequency by country for pediatrics-only trials (n = 3149). B, Heat map showing the number of pediatric-only clinical trials open for patient accrual in each country (n = 3149).

Characteristics of pediatrics-only clinical trials and combined trials are described in Table 1. Most trials were registered after 2010 (2194 of 3149 pediatrics-only trials [69.7%] and 3568 of 5645 combined clinical trials [63.2%]). Regionally, North America had the most sponsored studies (pediatrics-only trials, 1606 of 3149 [51.0%] and combined clinical trials, 2939 of 5645 [52.1%]), whereas Oceania had the fewest (pediatrics-only trials, 36 of 3149 [1.1%] and combined clinical trials, 78 of 5645 [1.4%]). Most pediatrics-only trials (2558 of 3149 [81.2%]) (Figure 1A) and combined clinical trials (4467 of 5645 [79.1%]) (eFigure 2 in Supplement 1) had sponsors in HICs. The most common countries of trial sponsors included the US (1548 of 3149 [49.2%]), followed by China (226 of 3149 [7.2%]) and Japan (177 of 3149 [5.6%]) (eTable 2 in Supplement 1). Primary trial sponsors were most frequently institutions (pediatrics-only trials, 2053 of 3149 [65.2%] and combined clinical trials, 3939 of 5645 [69.8%]). A collaborative group was the primary sponsor for 14.6% of pediatrics-only studies (459 of 3149). However, when secondary sponsors were included, collaborative groups sponsored 24.3% of pediatrics-only trials (766 of 3149). Among pediatrics-only trials, an equal number of studies accrued patients from a single site and multiple institutions (1493 of 3149 [47.4%]). For combined trials, most studies accrued patients from a single site (3108 of 5645 [55.1%]). For both groups, most trials accrued patients from a single country (pediatrics-only trials, 2465 of 3149 [78.3%] and combined clinical trials, 4750 of 5625 [84.2%]). Of the 3149 pediatrics-only clinical trials, 7 (0.2%) trials were open for accrual to patients from LICs, 281 (8.9%) from LMICs, and 394 (12.5%) from UMICs (Figure 1B). The countries with the highest number of trials available for patient accrual were the US (1605 of 3149 [51.0%]), Canada (404 of 3149 [12.8%]), Australia (295 of 3149 [9.4%]), France (262 of 3149 [8.3%]), and the UK (256 of 3149 [8.1%]), all of which are HICs (eTable 3 in Supplement 1).

**Table 1.** Demographic Characteristics of Pediatrics-Only and Combined Trials

Characteristic	Trials, No. (%)	
	Pediatrics only (n = 3149 [55.8%])	Combined (n = 5645 [100%])
Date registered		
1987-2010	954 (30.3)	2076 (36.8)
2011-2024	2194 (69.7)	3568 (63.2)
Registry source		
ClinicalTrials.gov	2293 (72.8)	4217 (74.7)
JPRN	167 (5.3)	330 (5.8)
EUCTR	159 (5.0)	208 (3.7)
ChiCTR	114 (3.6)	243 (4.3)
CTRI	104 (3.3)	148 (2.6)
IRCT	93 (3.0)	146 (2.6)
NL-OMON	63 (2.0)	71 (1.3)
ISRCTN	51 (1.6)	123 (2.2)
ANZCTR	37 (1.2)	78 (1.4)
DKRS	22 (0.7)	23 (0.4)
REBEC	15 (0.5)	19 (0.3)
CRIS	9 (0.3)	12 (0.2)
PACTR	8 (0.2)	9 (0.2)
TCTR	7 (0.2)	8 (0.1)
REPEC	3 (0.1)	6 (0.1)
CTIS	2 (0.1)	2 (0.04)
ITMCTR	1 (0.03)	1 (0.02)
RPCEC	1 (0.03)	1 (0.02)
Region of primary sponsor		
North America	1606 (51.0)	2939 (52.1)
Asia	729 (23.2)	1520 (26.9)
Europe	677 (21.5)	978 (17.3)
Africa	54 (1.7)	66 (1.2)
Latin America and Caribbean	47 (1.5)	64 (1.1)
Oceania	36 (1.1)	78 (1.4)
Primary sponsor country income status		
HIC	2558 (81.2)	4467 (79.1)
UMIC	329 (10.5)	494 (14.1)
LMIC	258 (8.2)	379 (6.7)
LIC	4 (0.1)	5 (0.1)
Primary sponsor type		
Institution	2053 (65.2)	3939 (69.8)
Collaborative group	459 (14.6)	604 (10.7)
Governmental organization	335 (10.6)	556 (9.8)
Pharmaceutical company	298 (9.5)	539 (9.6)
Other	4 (0.1)	7 (0.1)
Patient accrual sites		
Single	1493 (47.4)	3108 (55.1)
Multiple	1493 (47.4)	2206 (39.1)
Not mentioned	163 (5.2)	331 (5.8)
Single country	2465 (78.3)	4750 (84.2)
Multinational	684 (21.7)	895 (15.8)
Patient accrual country income status		
HIC	2544 (80.8)	4442 (78.7)
UMIC	394 (12.5)	906 (16.0)
LMIC	281 (8.9)	418 (7.4)
LIC	7 (0.2)	11 (0.2)

Abbreviations: ANZCTR, Australian New Zealand Clinical Trials Registry; ChiCTR, Chinese Clinical Trial Registry; CRIS, Clinical Research Information Service; CTIS, Clinical Trials Information System; CTRI, Clinical Trials Registry–India; DKRS, German Clinical Trials Register; EUCTR, EU Clinical Trials Register; HIC, high-income country; IRCT, Iranian Registry of Clinical Trials; ISRCTN, International Standard Randomized Controlled Trial Number; ITMCTR, International Traditional Medicine Clinical Trial Registry; JPRN, Japan Primary Registries Network; LIC, low-income country; LMIC, lower-middle-income country; NL-OMON, Overview of Medical Research in the Netherlands; PACTR, Pan African Clinical Trial Registry; REBEC, Brazilian Clinical Trials Registry; REPEC, Peruvian Clinical Trial Registry; RPCEC, Cuban Public Registry of Clinical Trials; TCTR, Thai Clinical Trials Registry; UMIC, upper-middle-income country.

Trial characteristics and inclusion criteria for both pediatrics-only trials and combined trials are described in Table 2. The highest percentage of trials was conducted for patients with solid tumors (pediatrics-only trials, 954 of 3149 [30.3%]; combined clinical trials, 1672 of 5645 [29.6%]) and hematologic malignant neoplasms (pediatrics-only trials, 882 of 3149 [28.0%]; combined clinical trials, 1884 of 5645 [33.4%]). There were similar numbers of frontline therapy trials (pediatrics-only trials, 1127 of 3149 [35.8%]; combined clinical trials, 1704 of 5645 [30.2%]) and trials for relapsed or refractory cancer (pediatrics-only trials, 1192 of 3149 [37.8%]; combined clinical trials, 2213 of 5645 [39.2%]). Cancer-directed treatments were the most common intervention type studied (pediatrics-only trials, 2221 of 3149 [70.5%]; combined clinical trials, 4170 of 5645 [73.9%]), followed by supportive care interventions (pediatrics-only trials, 828 of 3149 [26.3%]; combined clinical trials, 1271 of 5645 [22.5%]).

**Table 2.** Characteristics and Trial Inclusion Criteria for Pediatrics-Only and Combined Trials

Characteristic	Trials, No. (%)	
	Pediatrics only (n = 3149 [55.8%])	Combined (n = 5645 [100%])
Diagnoses		
Solid tumors	954 (30.3)	1672 (29.6)
Hematologic	882 (28.0)	1884 (33.4)
Central nervous system	490 (15.5)	587 (10.4)
Multiple tumor types	305 (9.7)	419 (7.4)
Bone marrow transplant	113 (3.6)	518 (9.2)
Molecularly defined	34 (1.1)	65 (1.1)
All cancers	371 (11.8)	500 (8.9)
Disease status		
Frontline	1127 (35.8)	1704 (30.2)
Relapsed or refractory	1192 (37.8)	2213 (39.2)
All statuses	434 (13.8)	851 (15.1)
Not mentioned	396 (12.6)	877 (15.5)
Type of intervention		
Treatment	2221 (70.5)	4170 (73.9)
Supportive care	828 (26.3)	1271 (22.5)
Diagnostic	100 (3.2)	204 (3.6)
Phase		
0	29 (0.9)	68 (1.2)
1	715 (22.7)	1190 (21.1)
1/2	314 (10.0)	636 (11.3)
2	848 (26.9)	1764 (31.2)
2/3	51 (1.6)	95 (1.7)
3	413 (13.1)	673 (11.9)
3/4	8 (0.3)	8 (0.1)
4	105 (3.3)	155 (2.8)
Not applicable	652 (20.7)	1024 (18.1)
Not mentioned	14 (0.4)	32 (0.6)
Published results		
Yes	1276 (40.5)	2237 (39.6)
No	1873 (59.5)	3408 (60.4)

**Figure 2.**
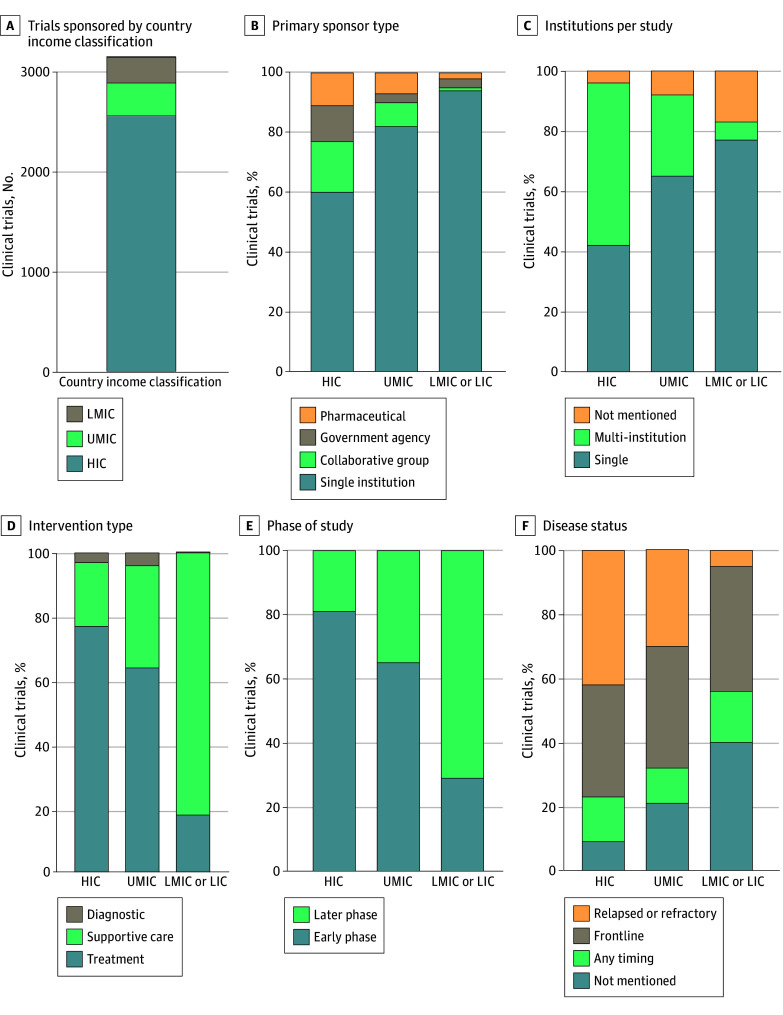
Clinical Trial Characteristics for Pediatrics-Only Trials by World Bank Country Classification A, Number of trials sponsored by country classification. B, Primary sponsor type. C, Number of institutions per study. D, Intervention type. E, Phase of study. F, Disease status. For all characteristics, *P* < .001 for upper-middle-income country (UMIC) and lower-middle-income country (LMIC) or low-income country (LIC) vs high-income country (HIC).

In pediatrics-only trials, trial demographics and characteristics differed by country income status (Figure 2; eTable 4 in Supplement 1). For all comparative analyses, *P* < .001. Trials sponsored by HICs had more variability in primary sponsor type, with a lower percentage of single-institution primary sponsors (1536 of 2558 [60.0%]) than trials in UMICs or in LMICs or LICs. HICs were also more likely to sponsor multi-institutional studies (1386 of 2558 [54.2%]) compared with UMICs and LMICs or LICs. Trials sponsored by HICs had more specific inclusion criteria and HICs were less likely to run clinical trials that enrolled patients with any cancer (235 [9.2%]) or trials that did not define a specific disease status (222 [8.7%]) compared with UMICs or with LMICs or LICs. Trials in HICs were more likely to be for a specific disease status, such as frontline (901 [35.2%]) or relapsed or refractory disease (1078 [42.1%]), whereas UMICs and LMICs or LICs were more likely to not mention disease status. Trials sponsored in UMICs and LMICs or LICs were more likely to be sponsored by a single-institution (UMICs, 270 of 329 [82.1%]; LMICs or LICs, 247 of 262 [94.3%]) than in HICs, with fewer trials that accrued from multiple institutions (UMICs, 90 of 329 [27.4%]; LMICs or LICs, 17 of 262 [6.5%]). Trials sponsored by HICs and UMICs were mostly treatment (HICs, 1964 of 2558 [76.8%]; UMICs, 211 of 329 [64.1%]) and early-phase (HICs, 1709 of 2100 [81.4%]; UMICs, 155 of 237 [65.4%]) trials; LMICs and LICs registered 46 of 262 treatment trials (17.6%) and 42 of 146 early-phase trials (28.8%). LMIC or LIC trials were primarily supportive care trials (214 of 262 [81.7%]) and later-phase studies (104 of 146 [71.2%]); HICs registered 509 of 2558 supportive care trials (19.9%) and 391 of 2100 later-phase studies (18.6%). Comparisons for combined trials were similar to those of pediatrics-only trials, with some exceptions (eTable 5 in Supplement 1).

**Figure 3.**
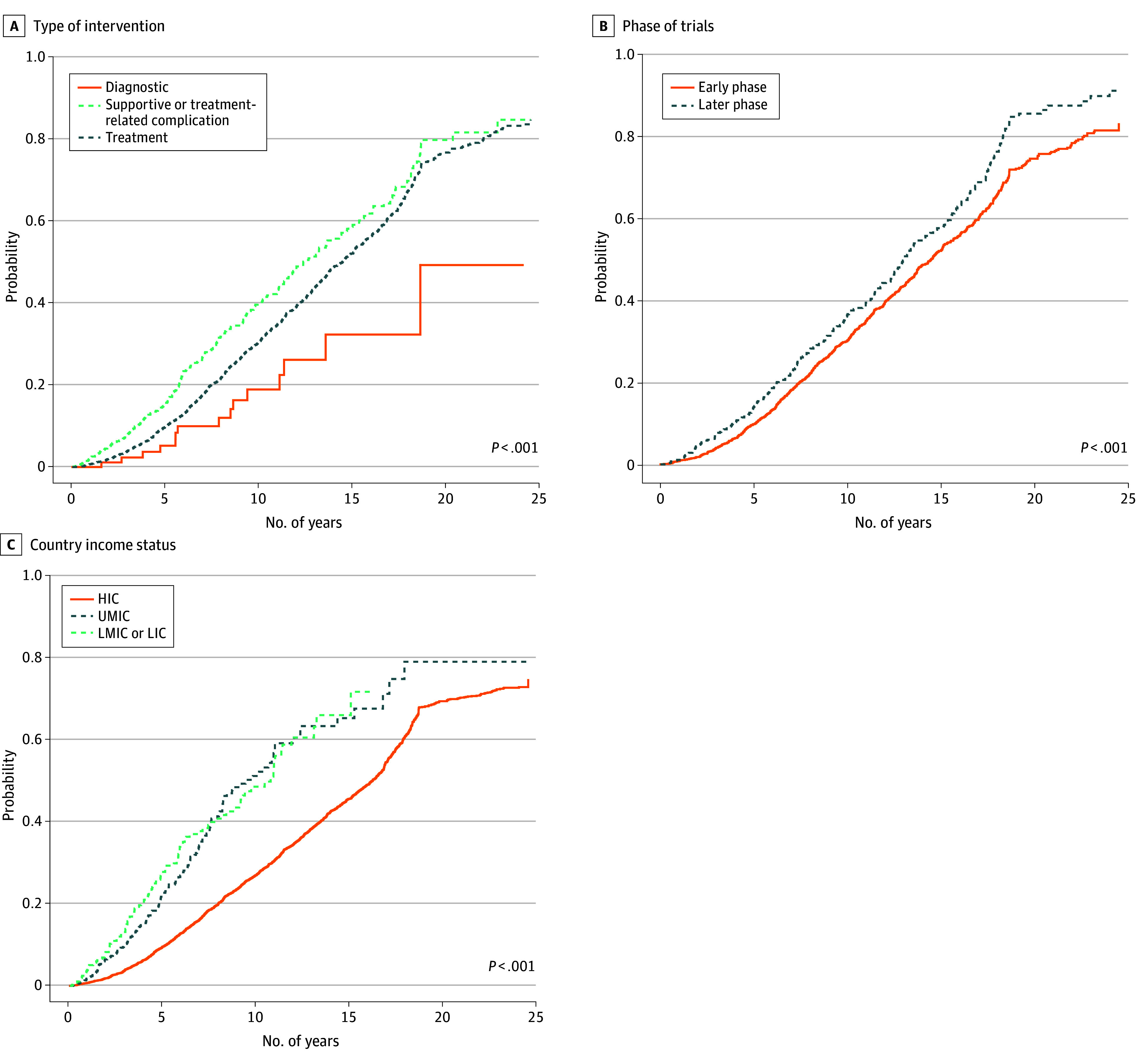
Cumulative Incidence Rate (Probability) of Peer-Reviewed Publications for Pediatrics-Only Trials From Their Date of Registration A, Type of intervention. B, Phase of trials. C, Country income status. HIC indicates high-income country; LIC, low-income country; LMIC, lower-middle-income country; and UMIC, upper-middle-income country.

Overall, 2237 of all 5645 studies (39.6%) and 1276 of the 3149 pediatrics-only trials (40.5%) had results published in a peer-reviewed manuscript. We compared publication rates by trial type and trial phase and across country income groups (Figure 3). At 10 years from the study registration, the mean (SD) publication rate was 34.9% (1.2%) for pediatrics-only trials evaluating cancer-directed treatment interventions, 43.2% (2.4%) for supportive care trials, and 18.9% (5.4%) for diagnostic trials (Figure 3A). When stratified by trial phase, early-phase trials had a 10-year mean (SD) publication rate of 35.0% (1.3%), while later-phase trials had a 10-year mean (SD) publication rate of 39.4% (2.5%) (Figure 3B). Based on country income group, the mean (SD) publication rates at 10 years were 27.1% (0.7%) in HICs, 51.1% (3.5%) in UMICs, and 48.5% (4.0%) in LMICs or LICs (Figure 3C). The median time to publication was 6.2 years (IQR, 3.5-11.0 years) for supportive care trials and 9.6 years (IQR, 6.1-14.0 years) for treatment trials. The median time to publication was 9.3 years (IQR, 5.8-14.0 years) for early-phase trials, and 9.3 years (IQR, 5.1-14.4 years) for later-phase trials. Based on income status, the median time to publication was 9.6 years (IQR, 6.9-14.0 years) for HICs, 4.2 years (IQR, 2.4-7.1 years) for UMICs, and 3.8 years (IQR, 2.1-5.9 years) for LMICs or LICs (*P* < .001). Cumulative incidence curves for combined trials are included in eFigure 3 in Supplement 2.

## Discussion

To our knowledge, this work provides the first global characterization of clinical trials for children and adolescents with cancer. These data show that low- and middle-income countries, particularly LMICs or LICs, are underrepresented in pediatric oncology clinical research. Not only are fewer studies registered or accruing patients in low- and middle-income countries but the existing studies more frequently involve supportive care rather than cancer-directed treatment. This underrepresentation is further amplified as most clinical studies in low- and middle-income countries are registered by a small number of countries.

The lack of pediatric oncology trials in low- and middle-income countries is likely multifactorial. Limited access to comprehensive diagnostics and novel therapeutics, along with a lack of funding and dedicated research staff, are often cited as reasons for less clinical research being conducted in these settings.^3^ In many low- and middle-income countries, patient to clinician ratios are high, and clinical care is often prioritized over academic obligations and research.^4^ Furthermore, although clinical trials have become the standard for pediatric cancer care in many HICs, there are still settings in which clinical trials are viewed as high-risk or exploitative toward vulnerable groups, leading to feelings of fear and mistrust.^19^ Diversity of cultural customs and norms can create unique regulatory, ethical, and enrollment challenges.^3^

Trials registered in HICs were more likely to be early-phase trials evaluating cancer-directed therapies with more granular inclusion criteria (ie, specific oncologic diagnoses or disease status). In comparison, trials registered in LMICs or LICs were later-phase studies, more likely to be focused on supportive care measures, and had more general inclusion criteria (ie, included all cancer diagnoses and/or did not mention specific disease status requirements). Patient populations in low- and middle-income countries are often at increased risk of treatment-related toxic effects due to higher rates of malnutrition, advanced disease at presentation, and comorbidities.^20^ This observation indicates that low- and middle-income countries are appropriately using the available clinical research infrastructure to develop improved and more reliable supportive care measures in these contexts. Supportive care trials are often more feasible in settings with limited diagnostic and therapeutic capacities, and are more cost effective than trials for novel cancer-directed treatments.^21^ Although the emphasis on supportive care studies in low- and middle-income countries may underscore gaps in research infrastructure and access to innovative treatments, it also reflects a strategic use of resources. By prioritizing supportive care, these contexts focus on interventions that yield the greatest improvement in childhood cancer care.

Single institutions were the most common primary sponsor type across all income statuses. However, there were more trials with collaborative groups or governmental sponsors in HICs than in low- and middle-income countries. In addition, collaborative groups sponsored a higher proportion of pediatric-only trials compared with combined clinical trials. This is probably a result of the relative rarity of childhood cancers and the need for multi-institution enrollment to accrue enough patients to achieve statistical and clinical significance in a timely manner.^22^ Childhood cancer care is improved in regions that have a collaborative group,^11^ suggesting that the development and strengthening of pediatric oncology collaborative groups in low- and middle-income countries may be a way to increase equity in pediatric oncology clinical research and improve childhood cancer care in these settings.

Given the relative lack of clinical trial availability in low- and middle-income countries, treatment strategies often involve resource-adjusted adaptations of the standard of care established by trials in HICs.^23^ However, results from clinical trials in high-income settings cannot be directly applied to low-resource settings. Patients in low- and middle-income countries can have different tumor biology, varying frequencies of germline mutations, comorbidities, a tendency toward more advanced disease, increased malnutrition, or other factors that can affect treatment toxic effects and efficacy.^6,8,9^ These differences can cause worse outcomes than those observed in HICs with the same therapies.^20^ This discrepancy reinforces the need to develop clinical trials designed by experts in low- and middle-income countries who understand the context of care in those settings. Furthermore, expanding the diversity of patient populations treated in clinical trials may improve our overall understanding of cancer biology and pharmacogenomics, ultimately leading to advances in the field of pediatric cancer.

We found that the median time to publication of clinical trials conducted in UMICs, LMICs, and LICs was shorter than for those in HICs. This discrepancy likely reflects the greater proportion of supportive care trials in UMICs, LMICs, and LICs. In contrast, trials from HICs were more frequently cancer-directed therapy trials, which may take more time to accrue adequate patient volumes, evaluate treatment efficacy, and analyze outcomes.^22,24^ These findings suggest that, despite having fewer clinical trials available, investigators in low- and middle-income countries are not only capable of conducting successful clinical research but are motivated to disseminate the results. This finding underscores the importance of investing in clinical trial infrastructure in all resource contexts.

### Limitations

This study has some limitations. First, it included only clinical trials registered to the ICTRP.^25^ Therefore, clinical trials not registered to one of these 18 primary registries were not captured. In addition, there are many countries in which recording a clinical trial registry is not mandatory. Registries included in the ICTRP must meet specific criteria, which helps to ensure quality and standardization and enabled us to obtain the same information from all included studies.^25,26^ The ICTRP is the most comprehensive clinical trial platform and is likely to be an adequate representation of the global clinical trial landscape. Second, our study did not include observational studies, which may be easier to conduct in resource-limited settings. However, beginning in 2005, the International Committee of Medical Journal Editors required the prospective registration of interventional studies as a condition for consideration of publication in a public trials registry.^27^ Similar policies have been implemented and expanded on by entities such as the European Commission and US Congress.^28^ However, there is a lack of policy and regulation surrounding noninterventional clinical studies and behavior-based interventional studies,^28^ which was cited as a reason to exclude noninterventional studies from a previous analysis.^29^ Third, with the characteristics readily available in the ICTRP database and in other publicly available resources, we were unable to assess the quality and biases of the included research studies. Future work should aim to evaluate the quality of research conducted across global regions.

## Conclusions

In this cross-sectional study of global pediatric oncology clinical trials, low- and middle-income countries were underrepresented in the pediatric oncology clinical landscape, despite having the highest burden of childhood cancer. The trials that did exist in low- and middle-income countries varied from those in HICs. Clinical trials can improve the quality of care and outcomes for pediatric cancer patients through new multimodal therapeutic approaches, improved risk stratification, and more robust supportive measures. Therefore, to advance childhood cancer care globally, more countries must have the capacity to perform high-quality research. Research capacity should be scaled up and scientific exploration should be adapted to fit the resource limitations and sociobiological factors. Collaborations that help provide this resource support in a culturally sensitive, mutually beneficial relationship are crucial. A multisectoral dialogue to improve research capacity is needed at the regional, national, and global levels. Developing new collaborations or enhancing existing collaborations can serve as a path for expanding research capacity globally.

## Notes

Supplement 1. **eFigure 1.** ICTRP Database Search and Study Selection **eTable 1.** Clinical Trials Used for Search Validation **eTable 2.** Frequency of Clinical Trial Sponsorship by Country **eTable 3.** Frequency of Clinical Trials Open for Accrual by Country **eFigure 2.** Geographic Distribution of Primary Sponsor and Patient Accrual for Clinical Trials **eTable 4.** Comparisons by World Bank Country Classification for Pediatrics-Only Trials **eTable 5.** Comparisons by World Bank Country Classification for Combined Clinical Trials **eFigure 3.** Cumulative Number of Peer-Reviewed Publications for Combined Trials From Their date of Registration

Supplement 2.
Data Sharing Statement
