# Supplementary material for: The Global Clinical Trial Landscape for Children and Adolescents With Cancer
Source: JAMA Netw Open. 2026 Jan 6;9(1):e2552510. doi: 10.1001/jamanetworkopen.2025.52510 (PMC12776201; doi:10.1001/jamanetworkopen.2025.52510)
Supplement: Supplement 2. — Data Sharing Statement [file jamanetwopen-e2552510-s002.pdf]

Data Sharing Statement

Mikkelsen. The Global Clinical Trial Landscape for Children and Adolescents With Cancer. *JAMA Netw Open*. Published January 06, 2026. doi:10.1001/jamanetworkopen.2025.52510

Data
Data available: Yes
Data types: Data (not involving human participants)
How to access data: Upon reasonable request to corresponding author.
When available: With publication

Supporting Documents
Document types: None

Additional Information
Who can access the data: To corresponding author (daniel.moreira@stjude.org)
Types of analyses: For any purpose.
Mechanisms of data availability: With investigator support.
