# Supplementary material for: The Global Clinical Trial Landscape for Children and Adolescents With Cancer
Source: JAMA Netw Open. 2026 Jan 6;9(1):e2552510. doi: 10.1001/jamanetworkopen.2025.52510 (PMC12776201; doi:10.1001/jamanetworkopen.2025.52510)
Supplement: Supplement 1. — eFigure 1. ICTRP Database Search and Study Selection eTable 1. Clinical Trials Used for Search Validation eTable 2. Frequency of Clinical Trial Sponsorship by Country eTable 3. Frequency of Clinical Trials Open for Accrual by Country eFigure 2. Geographic Distribution of Primary Sponsor and Patient Accrual for Clinical Trials eTable 4. Comparisons by World Bank Country Classification for Pediatrics-Only Trials eTable 5. Comparisons by World Bank Country Classification for Combined Clinical Trials eFigure 3. Cumulative Number of Peer-Reviewed Publications for Combined Trials From Their date of Registration [file jamanetwopen-e2552510-s001.pdf]

Supplementary Online Content

Mikkelsen MK, Pham LTD, Sotelo C, et al. The global clinical trial landscape for children and adolescents with cancer. *JAMA Netw Open.* 2026;9(1):e2552510. doi:10.1001/jamanetworkopen.2025.52510

eFigure 1. ICTRP Database Search and Study Selection
eTable 1. Clinical Trials Used for Search Validation
eTable 2. Frequency of Clinical Trial Sponsorship by Country
eTable 3. Frequency of Clinical Trials Open for Accrual by Country
eFigure 2. Geographic Distribution of Primary Sponsor and Patient Accrual for Clinical Trials
eTable 4. Comparisons by World Bank Country Classification for Pediatrics-Only Trials
eTable 5. Comparisons by World Bank Country Classification for Combined Clinical Trials
eFigure 3. Cumulative Number of Peer-Reviewed Publications for Combined Trials From Their date of Registration

This supplementary material has been provided by the authors to give readers additional information about their work.

© 2025 Mikkelsen MK et al. *JAMA Network Open.*

eFigure 1. ICTRP Database Search and Study Selection
Abbreviations: ICTRP, International Clinical Trials Registry Platform.

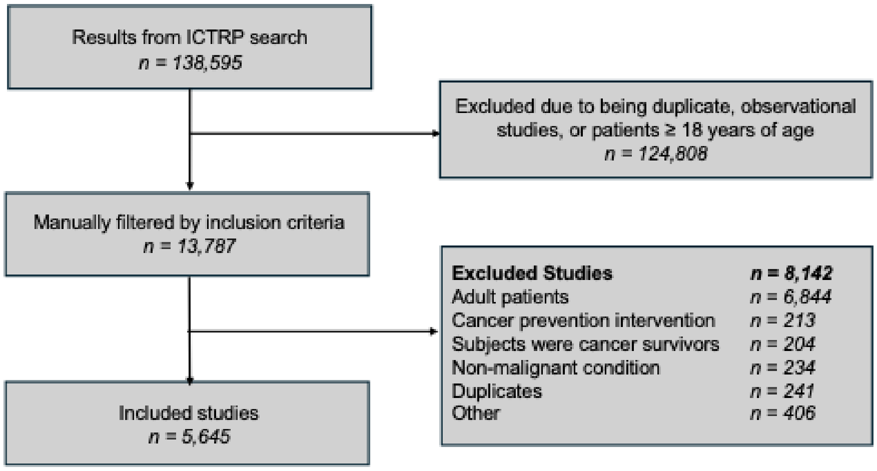

© 2025 Mikkelsen MK et al. *JAMA Network Open.*

eTable 1. Clinical Trials Used for Search Validation

Diagnosis	*HIC trials*			*LMIC trials*	
ALL	UKALL 2011 Trial ID - EUCTR2010-020924-22-GB	AALL0932 Trial ID - NCT01190930	Total XVII Trial ID - NCT03117751	Egypt NCT01656512	MASPORE Trial ID - NCT06336395
AML	AAML0531 Trial ID - NCT00372593				
Burkitt Lymphoma	ANHL01P1				
Hodgkin Lymphoma	AHOD1331 Trial ID - NCT02166463	cHOD17 Trial ID - NCT03755804			
Medulloblastoma	SJMB12 Trial ID - NCT01878617	ACNS1422 Trial ID - NCT00085735	SIOP-E Trial ID - EUCTR2011-004868-30-DE	Tata NCT04474964	
ATRT	SJATRT Trial ID - NCT02114229	ACNS0333 Trial ID - NCT00653068			
DIPG	SJPI3K Trial ID - NCT03696355			Egypt NCT01635140	
Pilocytic Astrocytoma	ACNS1833 Trial ID - NCT04166409				
Wilms Tumor	AREN0532 Trial ID - NCT00352534				
Neuroblastoma	ANBL0531- Trial ID - NCT00499616	ANBL15311- Trial ID - NCT03126916	NB2012 - Trial ID - NCT01857934	Argentina NCT01598454	Argentina NCT02998983
Osteosarcoma	EURAMOS-1 Trial ID - ISRCTN67613327				
Ewing Sarcoma	AEWS0031 Trial ID - NCT00006734				
Rhabdomyosarcoma	EpSSG EUCTR2005-000217-35-NO	ARST0431 Trial ID - NCT00354744	D9803 Trial ID- NCT00003958		
Retinoblastoma	ARET0321 Trial ID - NCT00554788				
Hepatoblastoma	AHEP1531 Trial ID - NCT03533582				
Germ cell tumors	SIOP GCT II Trial ID - EUCTR2009-018072-33-DE				

Abbreviations: ALL, acute lymphoblastic leukemia; AML, acute myeloid leukemia; ATRT, atypical teratoid/rhabdoid tumor; DIPG, diffuse intrinsic pontine glioma; HIC, high-income country; LMIC, lower middle-income country.

© 2025 Mikkelsen MK et al. *JAMA Network Open.*

eTable 2. Frequency of Clinical Trial Sponsorship by Country

Country	*Pediatrics-only trials (n)*	*Combined trials (n)*
United States	1,548	2,841
China	226	670
Japan	177	354
United Kingdom	137	268
France	122	164
India	156	108
Germany	97	112
Iran	96	155
Netherlands	76	84
Canada	57	97
Egypt	49	61
Italy	43	56
South Korea	43	70
Switzerland	43	55
Australia	36	77
Spain	30	45
Turkey	30	34
Brazil	28	36
Denmark	25	30
Sweden	24	32
Russia	19	22
Austria	17	19
Belgium	14	50
Hong Kong	14	14
Mexico	10	15
Israel	9	16
Poland	9	9
Thailand	7	8
Argentina	6	8
Taiwan	5	7
Finland	5	5
Indonesia	5	5
Singapore	4	17
Belarus	4	5
Czechia	4	4
Slovakia	4	4
Syria	2	2
Norway	1	6
Ireland	1	4
Saudi Arabia	1	4
Malaysia	1	3
Chile	1	2
Peru	1	2
Armenia	1	1
Bermuda	1	1
Cuba	1	1
Kenya	1	1

© 2025 Mikkelsen MK et al. *JAMA Network Open.*

Malawi	1	1
Nigeria	1	1
Philippines	1	1
Portugal	1	1
Tanzania	1	1
Uganda	1	1
Greece	0	2
Mongolia	0	1
New Zealand	0	1
Pakistan	0	1
Vietnam	0	1
Yemen	0	1

© 2025 Mikkelsen MK et al. *JAMA Network Open.*

eTable 3. Frequency of Clinical Trials Open for Accrual by Country

Country	***Pediatrics-only trials (n)***	***Combined trials (n)***
United States	1,605	2,930
Canada	404	509
Australia	295	399
France	262	379
United Kingdom	256	450
Netherlands	251	324
China	233	690
Germany	224	308
Italy	204	301
Japan	193	381
Spain	162	243
Switzerland	144	175
Puerto Rico	131	147
Denmark	118	149
India	117	177
Belgium	108	165
New Zealand	106	127
Austria	103	131
Sweden	99	124
Iran	96	155
Czechia	82	108
South Korea	72	120
Ireland	70	79
Israel	65	97
Norway	62	80
Poland	61	90
Brazil	53	83
Egypt	51	68
Finland	49	60
Russia	45	56
Turkey	40	56
Hungary	39	58
Portugal	37	58
Hong Kong	36	41
Argentina	27	40
Mexico	25	36
Greece	20	32
Slovenia	19	26
Singapore	18	47
Slovakia	18	37
Saudi Arabia	17	20
Chile	15	24
Taiwan	12	28
Thailand	10	13
Romania	9	13
Ukraine	8	9
Lithuania	7	11

© 2025 Mikkelsen MK et al. *JAMA Network Open.*

Belarus	7	8
South Africa	6	16
Croatia	6	15
Indonesia	6	7
Malaysia	5	8
Peru	5	8
Bulgaria	5	7
Iceland	5	5
Serbia	4	5
Colombia	3	3
Guatemala	3	3
Malawi	3	3
Uruguay	3	3
Estonia	2	4
Uganda	2	4
Kenya	2	3
Armenia	2	2
Cuba	2	2
Syria	2	2
Uzbekistan	2	2
Lebanon	1	2
United Arab Emirates	1	2
Ethiopia	1	1
Honduras	1	1
Jordan	1	1
Kyrgystan	1	1
Morocco	1	1
Nepal	1	1
Nigeria	1	1
Philippines	1	1
Swaziland	1	1
Tanzania	1	1
Vatican City	1	1
Zimbabwe	1	1
North Korea	0	2
Pakistan	0	2
Latvia	0	1
Malta	0	1
Mongolia	0	1
Panama	0	1
Vietnam	0	1
Yemen	0	1

© 2025 Mikkelsen MK et al. *JAMA Network Open.*

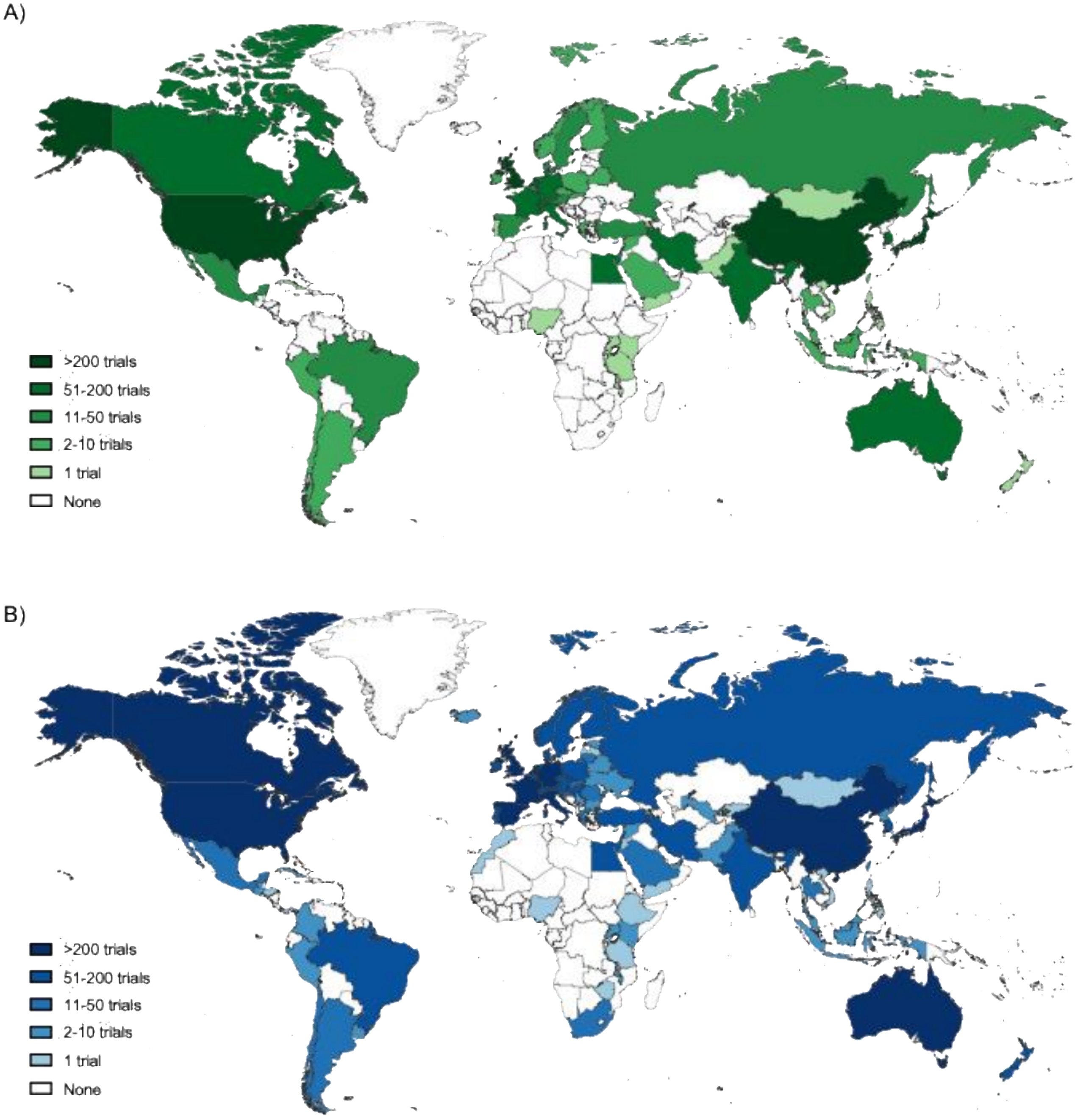

eFigure 2. Geographic Distribution of Primary Sponsor and Patient Accrual for Clinical Trials. (A) Heat map of primary sponsor frequency by country for combined trials. (B) Heat map of patient accrual frequency by country for combined trials.

© 2025 Mikkelsen MK et al. *JAMA Network Open.*

eTable 4. Comparisons by World Bank Country Classification for Pediatrics-Only Trials

	HIC<br>n (%)	UMIC<br>n (%)	LMIC/LIC<br>n (%)	Total<br>n	*P* value
**Total**	2558 (81)	329 (11)	262 (8)	3149	
**Primary Sponsor Type**					
Single institution	1536 (60)	270 (82)	247 (94)	2053	
Collaborative group	430 (17)	27 (8)	2 (1)	459	***<0.0001***
Government agency	316 (12)	10 (3)	9 (3)	335	
Pharmaceutical company	272 (11)	22 (7)	4 (2)	298	
Other	4 (0)	0 (0)	0 (0)	4	
**Patient accrual**					
Multi-institution	1386 (54)	90 (27)	17 (6)	1493	***<0.0001***
Single institution	1078 (42)	214 (65)	201 (77)	1493	
Not provided	94 (4)	25 (8)	44 (17)	163	
**Patient accrual**					
Single country	1882 (74)	322 (98)	261 (100)	2465	***<0.0001***
Multinational	676 (26)	7 (2)	1 (0)	684	
**Oncologic diagnoses**					
Solid tumor	818 (32)	100 (30)	36 (14)	954	
Hematologic	661 (26)	127 (39)	94 (36)	882	
Central nervous system	439 (17)	29 (9)	22 (8)	490	***0.0001***
Multiple tumor types	262 (10)	24 (7)	19 (7)	305	
Bone marrow transplant	109 (4)	2 (1)	2 (1)	113	
Molecularly driven	34 (1)	0 (0)	0 (0)	34	
Any cancer	235 (9)	47 (14)	89 (34)	371	
**Disease status**					
Frontline	901 (35)	124 (38)	102 (39)	1127	
Relapse/refractory	1078 (42)	102 (31)	12 (5)	1192	***0.0001***
All statuses	357 (14)	35 (11)	42 (16)	434	
Not mentioned	222 (9)	68 (21)	106 (40)	396	
**Type of intervention**					
Treatment	1964 (77)	211 (64)	46 (18)	2221	
Supportive care	509 (20)	105 (32)	214 (82)	828	***0.0001***
Diagnostic	85 (3)	13 (4)	2 (1)	100	
**Phase**					
Early phase	1709 (81)	155 (65)	42 (29)	1906	***0.0001***
Later phase	391 (19)	82 (35)	104 (71)	577	

Abbreviations: HIC, high-income country; LIC, low-income country; LMIC, lower middle-income country; UMIC, upper middle–income country.

© 2025 Mikkelsen MK et al. *JAMA Network Open.*

eTable 5. Comparisons by World Bank Country Classification for Combined Clinical Trials

	HIC n (%)	UMIC n (%)	LMIC/LIC n (%)	Total n	*P* value
**Total**	4467 (79)	794 (14)	384 (7)	5645	
**Primary Sponsor Type**					
Single institution	2903 (65)	674 (85)	362 (94)	3940	
Collaborative group	574 (13)	28 (4)	2 (1)	604	***<0.0001***
Government agency	532 (12)	12 (2)	12 (3)	556	
Pharmaceutical company	452 (10)	79 (10)	8 (2)	539	
Other	6 (0)	1 (0)	0 (0)	7	
**Patient accrual**					
Single institution	2244 (50)	572 (72)	292 (76)	3109	***<0.0001***
Multi-institution	2018 (45)	163 (21)	25 (7)	2206	
Not provided	205 (5)	59 (7)	67 (17)	331	
**Patient accrual**					
Single country	3583 (80)	785 (99)	382 (99)	4751	***<0.0001***
Multinational	884 (20)	9 (1)	2 (1)	895	
**Oncologic diagnoses**					
Solid tumor	1356 (30)	242 (30)	74 (19)	1672	
Hematologic	1363 (31)	396 (50)	125 (33)	1885	
Central nervous system	516 (12)	42 (5)	29 (8)	587	***<0.0001***
Multiple tumor types	354 (8)	37 (5)	28 (7)	419	
Bone marrow transplant	479 (11)	24 (3)	15 (4)	518	
Molecularly driven	65 (1)	0 (0)	0 (0)	65	
All cancers	334 (7)	53 (7)	113 (29)	500	
**Disease status**					
Frontline	1343 (30)	218 (27)	143 (37)	1704	
Relapse/refractory	1819 (41)	368 (46)	26 (7)	2213	***<0.0001***
All statuses	719 (16)	78 (10)	54 (14)	851	
Not mentioned	586 (13)	130 (16)	161 (42)	878	
**Type of intervention**					
Treatment	3476 (78)	600 (76)	94 (24)	4170	
Supportive care	831 (19)	155 (20)	285 (74)	1272	***<0.0001***
Diagnostic	160 (4)	39 (5)	5 (1)	204	
**Phase**					
Early phase	3123 (83)	462 (76)	73 (33)	3658	***<0.0001***
Later phase	638 (17)	143 (24)	150 (67)	932	

Abbreviations: HIC, high-income country; LIC, low-income country; LMIC, lower middle-income country; UMIC, upper middle–income country.

© 2025 Mikkelsen MK et al. *JAMA Network Open.*

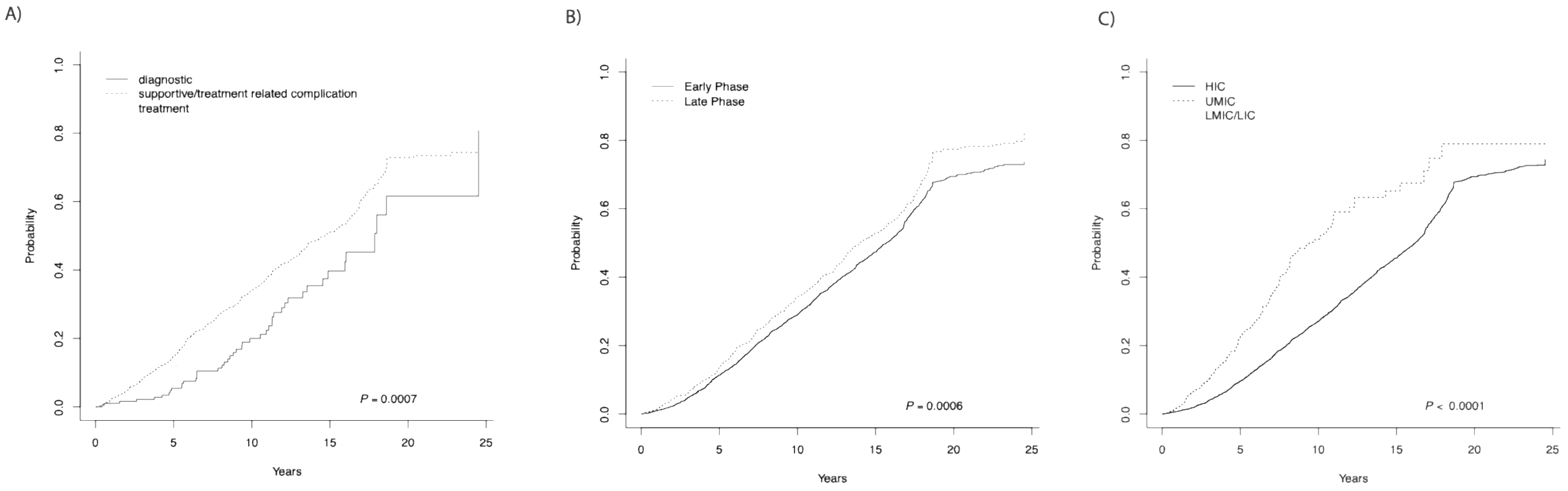

eFigure 3. Cumulative Number of Peer-Reviewed Publications for Combined Trials From Their date of Registration. Abbreviations: HIC, high-income country; LIC, low-income country; LMIC, lower middle–income country; UMIC, upper middle–income country.

© 2025 Mikkelsen MK et al. *JAMA Network Open.*
